# Chronic neuroinflammation impairs waste clearance in the rat brain

**DOI:** 10.3389/fnana.2022.1013808

**Published:** 2022-12-07

**Authors:** Swathi Suresh, Jacob Larson, Kenneth Allen Jenrow

**Affiliations:** ^1^Program in Neuroscience, Central Michigan University, Mount Pleasant, MI, United States; ^2^Department of Physics, Central Michigan University, Mount Pleasant, MI, United States; ^3^Department of Psychology, Central Michigan University, Mount Pleasant, MI, United States

**Keywords:** waste clearance, glymphatic, IPAD, neuroinflammation, lipopolysaccharide, amyloid β, Alzheimer’s disease

## Abstract

**Background:**

Previous reports have established an association between impaired clearance of macromolecular waste from the brain parenchyma and a variety of brain insults for which chronic neuroinflammation is a common pathological feature. Here we investigate whether chronic neuroinflammation is sufficient to impair macromolecular waste clearance from the rat brain.

**Methods:**

Using a rodent model of chronic neuroinflammation induced by a single high-dose injection of lipopolysaccharide, the clearance kinetics of two fluorophore-conjugated dextran tracers were assayed at 8-weeks post-induction. The expression and distribution of amyloid β and aquaporin-4 proteins within selected brain regions were assayed at 36-weeks post-induction, following open-field, novel object recognition, and contextual fear conditioning assays.

**Results:**

Chronic neuroinflammation significantly impaired the clearance kinetics of both dextran tracers and resulted in significantly elevated levels of amyloid β within the hippocampus. Aquaporin-4 density on astrocytic endfeet processes was also reduced within multiple brain regions. These pathologies were associated with significantly enhanced contextual fear memory.

**Conclusion:**

Our results suggest that chronic neuroinflammation is sufficient to compromise the clearance of macromolecular waste from the brain parenchyma and may be the root cause of impaired waste clearance associated with a variety of brain pathologies.

## Introduction

Chronic neuroinflammation is frequently associated with dramatic reductions in the clearance of macromolecular waste from the brain parenchyma. Both chronic neuroinflammation and impaired clearance are common pathological features associated with Alzheimer’s disease, traumatic brain injury (TBI), and stroke, and are also present in the aged brain, suggesting that there may be a causal relationship between them (Sabbatini et al., 1999; Ross and Poirier, 2004; Kress et al., 2014; Heppner et al., 2015; Plog et al., 2015; Simon and Iliff, 2016; Stephenson et al., 2018; Zhang et al., 2022). Activated microglia play a prominent role in initiating neuroinflammation and are the primary source of proinflammatory cytokines such as tumor necrosis factor α (TNFα), interleukin 6 (IL-6), and interleukin 1β (IL-1β) (Liddelow et al., 2017). Sustained release of these cytokines commonly facilitates activation of adjacent astrocytes, which amplify the inflammatory response *via* additional cytokine release (Godbout et al., 2005; Hayakawa et al., 2007; Li et al., 2009; Lyons et al., 2009; Liddelow et al., 2017). Chronic glial activation and release of these proinflammatory cytokines may be the most important etiological factors in age-related neurodegenerative diseases and are commonly present long before the associated neuropathologies and neurological symptoms become detectable (Ceulemans et al., 2010; Khandelwal et al., 2011). Here we investigate the hypothesis that chronic neuroinflammation, in the absence of other pathologies, may be sufficient to cause a reduction in the clearance of macromolecular waste from the brain parenchyma.

The brain parenhyma lacks lymphatic vessels and it has long been recognized that the clearance of macromolecular waste from the parenchyma must occur by other means (Cserr and Ostrach, 1974; Cserr et al., 1977; Rennels et al., 1985, 1990; Louveau et al., 2015). A consensus has emerged within the past decade that waste clearance from the brain parenchyma is facilitated by a dynamic exchange between cerebrospinal fluid (CSF) and interstitial fluid (ISF). Compelling evidence has accrued in support of two hypothesis involving perivascular CSF/ISF transport. The glymphatic system hypothesis proposes that subarachnoid CSF infiltrates the brain by coursing through periarterial space, driven by cardiac pulsations, and enters the parenchyma by passing through aquaporin-4 (AQP4) channels embedded in astrocytic endfeet processes (the glia limitans). CSF mixes with ISF and conducts waste products through interstitial space by convective bulk flow toward perivenous spaces, where they are collected and transported out of the parenchyma (Iliff et al., 2012, 2013, 2014; Xie et al., 2013; Kress et al., 2014; Zeppenfeld et al., 2017; Silva et al., 2021). The intramural periarterial drainage (IPAD) hypothesis proposes that subarachnoid CSF infiltrates the brain by coursing through arterial pial-glial basement membranes, driven by cardiac pulsations, and enters the parenchyma by passing through the glia limitans. CSF mixes with ISF and promotes the diffusion of interstitial waste products toward the capillaries, where they enter capillary basement membranes and are transported out of the parenchyma by vascular smooth muscle cell contractions within the arterial tunica media (Keable et al., 2016; Morris et al., 2016; Smith et al., 2017; Albargothy et al., 2018; Aldea et al., 2019; Van Veluw et al., 2020). There are inconsistencies between these two hypotheses regarding the importance of aquaporins for CSF entry into the parenchyma, whether waste products move by convective bulk flow or diffusion within interstitial space, and whether waste products are cleared within perivenous space or periarterial basement membranes. These reflect disparities among numerous reports supporting these two hypotheses that have generally been attributed to methodological differences; however, critical issues remain unresolved.

A recent report by Alshuhri et al. (2021) strongly supports a role for AQP4 in facilitating CSF entry into the brain parenchyma, as predicted by the glymphatic hypothesis. Using magnetic resonance imaging (MRI), they visualized the movement of isotopic water molecules infused into subarachnoid CSF and found that water entry into the paranchyma was reduced by approximately 80 percent when rats were pretreated with an AQP4 inhibitor. Zeppenfeld et al. reported that reduced AQP4 channel polarization on astrocytic endfeet processes in the aged brain similarly impaired the entry of dextran tracers into the parenchyma and was associated with a 40 percent decrease in the clearance of intraparenchymally injected amyloid β (Aβ) (Kress et al., 2014; Zeppenfeld et al., 2017). Reduced AQP4 channel polarization has also been reported in Alzheimer’s disease, and following TBI and stroke, suggesting that a resultant decrease in CSF entry into the parenchyma may contribute to impaired waste clearance associated with these pathologies (Khandelwal et al., 2011; Yang et al., 2011, 2022; Zeppenfeld et al., 2017).

Here we adopted a rodent model of chronic neuroinflammation to investigate whether macromolecular clearance is impaired in the chronically inflammed brain. A single high-dose injection of lipopolysaccharide (LPS) is used to produce a transient systemic inflammatory response accompanied by elevated expression of TNFα. Circulating TNFα rapidly crosses the blood-brain barrier (BBB) in a TNFα-receptor (TNFR)-dependent manner, where it produces a parallel inflammatory response within the brain characterized by microglial activation and elevated levels of TNFα, IL-1β, IL-18, MCP-1, and NF-κB. Remarkably, the peripheral inflammatory response subsides within hours whereas chronic neuroinflammation is fully established within 7 days and persists unabated for more than 10 months (Qin et al., 2007; Bossù et al., 2012). An identical chronic neuroinflammatory response can be induced by a single intravenous injection of TNFα, at concentrations comparable to those produced by high-dose LPS (Qin et al., 2007). In the mouse brain, this chronic neuroinflammatory resonse is associated with a progressive loss of dopaminergic neurons within the substantia nigra; however, this is a delayed response requiring more than 24-weeks to become detectable.

To test our hypothesis, we assayed the clearance kinetics of two flurophore-conjugated dextran tracers following 8-weeks of chronic neuroinflammation, well in advance of any putative neurotoxic effects. Cognitive function assays were performed between 30- and 36-weeks after initiating chronic neuroinflammation, when we anticipated that the cumulative effects of impaired waste clearance might be detectable. These behavioral assays were followed by qualitative and quantitative measures of AQP4 and Aβ protein expression in the hippocampus and pre-frontal cortex. Our results demonstrate for the first time that chronic neuroinflammation is sufficient to impair macromolecular clearance from the brain, and identify chronic neuroinflammation as a promising therapeutic target for preventing or mitigating clearance disorders.

## Materials and methods

### Experimental design

All experiments were approved by the Central Michigan University Institutional Care and Use Committee. Forty-four, male Sprague–Dawley rats were obtained commercially from (Charles River Laboratories, Wilmington, MA, USA) and were pair-housed in a 12:12 h reverse light cycle room, with food and water provided *ad libitum*. At least 2 weeks were allowed for acclimation to these housing conditions before initiating experimental work.

**FIGURE 1:**
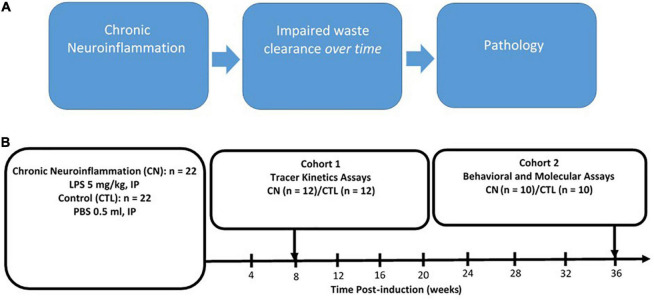
Rationale and experimental design. **(A)** Rationale for chronic neuroinflammation as an initiator of chronic neuroinflammation and impaired waste clearance. **(B)** Experimental design: chronic neuroinflammation was induced in male Sprague–Dawley rats by a single intraperitoneal injection of lipopolysaccharide (5 mg/kg, i.p.) (CN, *n* = 22), with Control group (CTL) rats receiving equivalent injections of phosphate buffered saline (∼0.5 ml, i.p.) (CTL, *n* = 22). At 8-weeks post-induction, all Cohort 1 rats (CN, *n* = 12; CTL, *n* = 12) received infusions of fluorophore-conjugated dextran tracers into the subarachnoid CSF, and tracer kinetics were assayed following circulation times of 15-, 30-, and 45-min post-infusion (*n* = 4 per subgroup). Between 30- and 36 weeks post-induction, all Cohort 2 rats (LPS, *n* = 10; CTL, *n* = 10) were subjected to behavioral assays (open-field, novel object recognition, and fear conditioning) and sacrificed at 36-weeks for protein analysis using immunofluorescence and Western blot.

The experimental design is shown in Figure 1. At approximately 12 weeks of age, rats were randomly assigned to either Chronic Neuroinflammation (CN) (*n* = 22) or Control (*n* = 22) groups. The CN group received single injections of LPS (5 mg/kg, i.p.), whereas the Control group received single injections of equivalent volumes of sterile phosphate buffered saline (PBS, 0.5 ml, i.p.). Rats in both groups were randomly assigned to one of two cohorts: Cohort 1 rats (CN, *n* = 12; Control, *n* = 12) received 10-μL infusions of tracer solution delivered to the cisterna magna after 8 weeks of chronic neuroinflammation. Separate subgroups (*n* = 4 per subgroup) were sacrificed following circulation intervals of 15-, 30-, and 45-min post-infusion, respectively, to assay the kinetics of tracer distribution and clearance. Upon completing their respective circulation intervals, 1 ml of whole blood was withdrawn from the left cardiac ventricle and rats were transcardially perfused with PBS followed by paraformaldehyde. Cohort 2 rats (CN, *n* = 10; Control, *n* = 10) received cognitive function assays between 30- and 36-weeks of chronic neuroinflammation, using the open-field, novel object recognition, and contextual fear conditioning assays. These rats were subsequently sacrificed by either transcardial perfusion for immunofluorescence (*n* = 5 per subgroup) or by rapid decapitation for protein quantification by Western blot (*n* = 5 per subgroup) (Figure 1).

### Lipopolysaccharide administration

All rats weighed between 250 and 300 g at the time of LPS administration. LPS (*E. coli* strain 055:B5, Sigma-Aldrich, St. Louis, MO, USA) was freshly dissolved in sterile PBS at a concentration of 5 mg/ml and administered by intraperitoneal injection at a dose of 5 mg/kg. At this dose, LPS reliably causes acute systemic inflammation and rats exhibit “sickness behavior,” characterized by fever, reduced appetite, and lethargy (Bossù et al., 2012). Sickness behavior generally peaked within 1–2 h and subsided within 24 h post-injection. Rats were closely monitored during this interval and were provided with supplemental fluids when necessary. All rats fully recovered by 48-h post-injection. Previous reports have established that systemic levels of TNFα and other proinflammatory cytokines return to normal within 24 h post-LPS (Qin et al., 2007). Remarkably, however, the transient spike in serum TNFα triggers a chronic inflammatory response within the brain that persists unabated for at least 10-months post-injection (Qin et al., 2007; Bossù et al., 2012).

### Intracisternal cerebrospinal fluid tracer infusion

A 200-μl solution containing Texas-red conjugated 3-kDa dextran and FITC conjugated 10-kDa dextran tracers, each at concentrations of 0.5% in aCSF, was freshly prepared prior to initiating surgeries. Dextran tracers are widely used for clearance studies because they are not readily cleared by direct transport across the BBB or by cellular uptake (Iliff et al., 2012, 2013, 2014; Xie et al., 2013; Albargothy et al., 2018). Rats (CN, *n* = 12; CTL, *n* = 12) were anesthetized with ketamine (80 mg/kg, i.p.) and xylazine (10 mg/kg, i.p.) and mounted in a stereotactic frame (Koph Instruments, Tujunga, CA, USA). The occipital bone and the atlanto-occipital membrane were surgically exposed by making a midline incision and retracting the overlying muscle. A Hamilton syringe attached to a beveled 27-gauge needle was preloaded with 15 μl of tracer solution. The syringe was placed in a syringe pump (KD Scientific, Holliston, MA, USA) and the tip of this needle was advanced into the cisterna magna, extending approximately 2.3 mm beyond the atlanto-occipital membrane. After a 1-min delay, the syringe pump was activated to infuse 10 μl of tracer solution into the cisterna magna at a rate of 2 μl/min. The needle was left in place throughout the designated circulation interval to maintain intracranial pressure and to prevent tracer efflux. To visualize the distribution kinetics of these tracers, circulation intervals of 15-, 30-, and 45-min were used for three separate subgroups of animals (*n* = 4 CN and *n* = 4 Control per subgroup), with each of these circulation intervals including the 5-min infusion interval. At the end of the circulation interval, the needle was rapidly withdrawn from the cisterna magna, and the rat was immediately removed from the stereotaxic apparatus for rapid transcardial perfusion with ice-cold PBS followed by paraformaldehyde (PFA). Immediately prior to perfusion, 1 mL of whole blood was drawn directly from the left cardiac ventricle from which the serum fraction was isolated by centrifugation and frozen at −80°C for later analysis. Perfused brains were immediately removed from the skull and post-fixed in PFA for 24 h at 4°C. Cryopreservation consisted of successive equilibrations in 15 and 30% sucrose solutions, after which brains were flash-frozen in isopentane at −40°C, and stored at −80°C until use.

### *Ex vivo* florescence imaging and analysis

Brains were vibratome-sectioned in the coronal plane at 100 μm. Approximately 10–12 sections were acquired per animal and slide-mounted with Antifade Gold mounting medium with DAPI (Life Technologies, Carlsbad, CA, USA). Tracer penetration within the parenchyma was visualized within these sections using *ex vivo* epifluorescence microscopy. Multi-channel whole-slice montages were acquired at 5X on the Zen Blue 2.3 software (Carl Zeiss AG; Oberkochen, Germany), including separate channels for DAPI (blue), FITC (green), and Texas- red (red). Exposure levels were maintained constant throughout this acquisition process at 30-, 90-, and 150-ms for excitation wavelengths at 358-, 488-, and 594-nm, respectively. Sections were traced using the DAPI-channel images and Z-stacks were collected from five successive focal planes at 10-μm intervals, acquired using the 20X objective. Images were flattened using an extended depth of focus, and the tiled images were stitched and exported. Quantification of tracer distribution within each section was performed using ImageJ software (NIH), using a modified version of the thresholding protocol described by Iliff et al. (2012).

### Tracer quantification

Quantification of tracer distribution within images acquired from coronal brain sections was performed using the densitometry analysis functions of ImageJ software (NIH). A calibration slide (Slide F36909, row A, Thermo Fisher Scientific, Rockland, IL, USA) was used prior to image acquisition to check for chromatic aberrations by switching between channels and verifying x/y alignment and the accurate overlay of channels in the red, green, and blue wavelength. All images were analyzed using the Bioformat importer plug-in feature of ImageJ to preserve bit depth and maximize signal. Images were converted to 8-bit and the ROI manager was used to select the entire section, using DAPI as a reference. The background value was recorded separately for each channel in each section. This value was then subtracted from each corresponding channel, after which the sections were thresholded. Based on previously published reports (Iliff et al., 2012), threshold levels were set at 75 for both FITC and Texas-Red channels, and the total area, mean OD, and the area fraction above threshold was recorded for each channel in every section. Tracer coverage for each section was expressed as the percent of the total section area above threshold. Mean values of tracer coverage for each animal were calculated by averaging the tracer coverage across sections and expressed as a percentage of the total area.

### Spectrophotometry

Whole blood samples (1 ml) drawn from the left ventricle immediately prior to transcardial perfusion were immediately transferred to 1-ml microcentrifuge tubes and centrifuged for 15 min to isolate the serum fraction. Serum was then pipet-transferred to a 1-ml cryovial and stored at −80°C. Samples were thawed and brought to room temperature and serum aliquots (200 μl) were transferred to an opaque black 96-well microtiter plate, for analysis using an M2 multi-channel fluorescence spectrophotometer (Molecular Devices, San Jose, CA, USA). A series of dilution standards of known concentrations were prepared using PBS for both the 3-kDa and 10-kDa dextran tracers, and aliquoted in 200-μl volumes in separate rows on the same 96-well microtiter plate. The concentrations used for each of these tracers were 5-, 10-, 25-, and 50-ηg/μl. Fluorescence values associated with each of these concentrations were graphed and linear trend-lines with zero intercepts were fitted to the points for each tracer. Slopes for each of these trend-lines were used to express the fluorescence values associated with each tracer obtained from our serum samples as a concentration in units of ng/μl.

### Behavioral assessment

Behavioral assays were performed between 30 and 36 weeks after LPS-induced chronic neuroinflammation to investigate whether chronic neuroinflammation and/or impaired waste clearance was associated with cognitive impairment. These assays were performed on all Cohort 2 rats (CN, *n* = 10; Control, *n* = 10) and were sequenced in order of increasing stress induction, with a minimum of 2 days allowed for recovery between each assay. The open-field assay was administered first as a measure of general anxiolytic effects, followed by novel object recognition as a measure of recognition memory, and contextual fear conditioning as a measure of fear-induced contextual memory.

#### Open-field

The open-field assay is commonly used as a measure of both general anxiety and locomotor activity (28). Our open-field assays were performed using a SmartFrame system with system performance and data collection controlled by MotorMonitor software (Kinder Scientific, Chula Vista, CA, USA). Rats were placed individually into one of eight transparent Plexiglas boxes (44 cm × 44 cm × 38 cm), each surrounded by a SmartFrame with the upper and lower frames positioned 4 cm and 10 cm, respectfully, above the floor of the box. At the beginning of the trial, each rat was placed in the center of the box and recording was initiated immediately thereafter. The rat was allowed to roam freely within the box for 1 h, during which its movements within the box were recorded using a zone map appropriate to the size of the rat. Zones used for analysis, defined as center and periphery, were recorded in variables of time spent in different zones. The total distance traveled was also measured. Light intensity was 30 lux during these recording sessions, as measured within the center of the boxes at floor level. The percentage of time spent in the center and periphery zones was interpreted as a measure of anxiety, and the total distance traveled was interpreted as a measure of arousal and general motor function (Seibenhener and Wooten, 2015).

#### Novel object recognition

Rodents have a natural proclivity for exploring novel, relative to familiar, and objects. The novel object recognition (NOR) assay takes advantage of this selective preference to test recognition memory. The apparatus used for our NOR assay consisted of a custom-built rectangular chamber (76 cm × 56 cm × 30 cm) with an open roof. The chamber was made of black opaque polycarbonate, which was abraded to reduce reflectivity. The chamber was surrounded by a black curtain suspended from the ceiling and the chamber floor was uniformly illuminated from above at a light intensity of five lux. Animal behavior was recorded using a video camera suspended 79 cm above the center of the chamber. Prior to initiating the experiment, we performed a preliminary study using a separate group of animals to test for any inherent biases related to the objects employed or to left or right sides of the chamber. The assay was performed on consecutive days in three phases: Habituation, acquisition, and testing, respectively, with all phases conducted during the last 4 h of the active cycle of the rats. Videos were recorded throughout these three phases for offline analysis. On Day 1, habituation was performed to familiarize rats with the transfer process, the chamber with no objects present, and to the video camera. Rats were allowed to freely explore the chamber for 15 min and were then returned to their cages. On Day 2, the acquisition phase, two identical objects (Objects A1 and A2) were present in the chamber and rats were allowed to freely explore them in a single 15-min trial. These identical objects (inverted ceramic bowls) were placed in the left and right rear corners of the chamber, at a distance of 3 cm from the rear and side walls. After completing the trial, rats were returned to their home cages. On Day 3, the testing phase, one of the identical objects was replaced by a “novel” object (an inverted cylindrical glass jar), representing Object B, while the other, now-familiar object was replaced by Object A3, identical in all aspects to Objects A1 and A2, and rats were allowed to explore the chamber for 10 min. During all phases, the chamber and objects were thoroughly cleaned with 70% ethanol and allowed to dry in between trials to eliminate olfactory cues. Object positions were counterbalanced between groups to avoid any side bias.

Exploration was operationally defined having the head oriented toward the object with the nose within a 2-cm perimeter with vibrissae movement (Clark et al., 2000), and was scored offline by an experimenter blind to group identification. Climbing over the objects, using the objects as platform, or any other type of incidental contact with the objects was not scored as exploration. The amount of time each rat spent exploring the novel object [*t* (OBJECT B)] and the familiar object [*t* (OBJECT A3)] were recorded using these criteria. Preference for the novel object was expressed as a “Recognition Index” (RI), defined as the percentage of time spent exploring the novel object relative to the total time spent exploring both objects.


RI=t(OBJECTB)/[t(OBJECTA3)+t(OBJECTB)]×100.

#### Contextual fear conditioning

The contextual fear-conditioning assay measures the recall of an association between an unconditioned stimulus (a mild foot shock) and a conditioned stimulus (a chamber with specific contextual features). All contextual fear conditioning was performed in a specifically designed fear conditioning system (Kinder Scientific, Chula Vista, CA, USA). The shock chamber was made of clear Plexiglas, and measured approximately 43 cm × 23 cm × 20 cm. The roof of the chamber was removable and was also clear Plexiglas with holes to allow for ventilation. During testing, two video cameras were used to film animal behavior, one mounted above the chamber and the other mounted laterally, to get a comprehensive view of the animal from nose to tail. Animal behavior was recorded using the SmartFrame system (Kinder Scientific, Chula Vista, CA, USA), with the lower frame mounted 4 cm and the upper frame mounted 10 cm above the cage floor. System performance and data collection were controlled by MotorMonitor software (Kinder Scientific, Chula Vista, CA, USA). Context A consisted of an exposed shock grid on the floor of the chamber, a transparent ceiling, and opaque panels covering the back wall and the two sidewalls. Context B consisted of a striped plastic panel, which covered the shock grid on the floor of the chamber, a transparent ceiling with ventilation holes, and opaque panels covering the front and black walls.

Exposure to both contexts occurred in the same room with all other features maintained consistently. On Day 1, rats were placed in a holding area outside the testing room and transferred individually to Context A. To facilitate context association, 3 min of exploration was allowed before the shock was delivered. The shock was administered through the floor grid as a single 2-s duration pulse train, at a constant current of 1.2 mA. Two minutes after receiving the shock, rats were removed from Context A and returned to their home cages. On Day 2, rats were placed in a holding area, and each rat was placed in Context A and allowed to explore freely for 10 min with no additional shock delivered during this interval. Finally, on Day 3, animals were placed in Context B and allowed to roam freely for 10 min. Two video cameras and the SmartFrame system recorded behavior during each of these sessions and the duration of freezing behavior was subsequently analyzed offline (Poulous et al., 2016; Chaaya et al., 2018).

### Immunofluorescence

After completing the behavioral assays, Cohort 2 subgroups (CN, *n* = 6; CTL, *n* = 5) were processed for immunofluorescence. Rats were deeply anesthetized with isoflurane and transcardially perfused, first with ice cold PBS followed by 4% paraformaldehyde. Brains were immediately removed from the skull and post-fixed in PFA for 48 h at 4°C, after which they were equilibrated using a sucrose gradient, first at 15 and then 30%, for cryoprotection. Brains were then flash-frozen in 2-methylbutane (Sigma-Aldrich, St. Louis, MO, USA) at −40°C and stored at −80°C until further processing. Immunofluorescence was conducted on free floating, 30-μm cryostat sections. Slices were blocked for 1 h with 1% BSA at room temperature primary antibody incubation in TBS with 0.1% Triton X-100, overnight at 4°C with gentle agitation. Sections containing the hippocampus and pre-frontal cortex were incubated with primary antibodies against Aβ (Rabbit anti-Aβ, AB10148; 1:200, Abcam, Cambridge, UK) on separate slides, and double-labeled for AQP4 (Rabbit anti- AQP4, AB3594; 1:1,000, Abcam, Cambridge, UK), and GFAP (Mouse anti-GFAP, MAB3402; 1:1,000, Millipore Sigma, Burlington, MA, USA) (LPS, *n* = 6; CTL, *n* = 5). Secondary antibodies (anti-rabbit and anti-mouse, Invitrogen, Waltham, MA, USA) were added for 1 h at room temperature. Sections were then rinsed three times in Tris-buffered saline with 0.1% Tween-20 (TBST). Finally, slices were mounted on positively charged slides with Prolong Antifade Gold with DAPI (Life Technologies, Carlsbad, CA, USA).

### Western blot

After completion of behavioral assays, separate Cohort 2 subgroups (CN, *n* = 4; CTL, *n* = 4) were processed for semi-quantitative analysis of the proteins AQP4 and Aβ 1–42 using Western blot, using established protocols (Al-Gharaibeh et al., 2017). Rats in these subgroups were deeply anesthetized with isoflurane and decapitated. Brains were immediately removed from the skull and flash frozen in 2-methyl-butane at −40°C and stored at −80°C. Brain tissue was equilibrated to −20°C and tissue from the hippocampus and pre-frontal cortex was dissected from multiple sections from each brain and homogenized. Cells were lysed in radio-immuno-precipitation assay (RIPA) buffer containing 10-mM Tris-Cl,1-mM EDTA (metal chelator), 1% Triton X-100, 0.1% sodium deoxycholate,0.1% SDS, and 140-mM NaCl, along with a cocktail of protease inhibitors (Sigma-Aldrich, St. Louis, MO, USA). After centrifuging at 20 *g* at 4°C for 30 min, supernatant was aliquoted in 20-μl tubes and stored at −80°C until use, with the exception of one tube each that was used for protein estimation. Protein concentrations across samples were calculated using the Pierce BCA protein assay (Thermo Fisher Scientific, Rockland, IL, USA). This assay relies on the selective, calorimetric detection of reduced cuprous cations in bicinchoninic acid in an alkaline medium, the reduction being a function of the protein concentration. Aliquots of 20 μl were mixed with equal amounts of 2X SDS sample buffer containing 100-mM Tris-HCL, pH 6.8, 20 glycerol, 10 2-mercaptoethanol, and 0.2 bromophenol blue and boiled for 5 min. After protein quantification, 40 ug of protein was loaded into each well and separated using SDS-PAGE on a 4–20% gradient SDS gel at 100V. Proteins were then transferred overnight to a PVDF membrane in a transfer buffer at 4C. Staining with 0.1% Ponceau was used to verify protein transfer, after which the membranes were washed three times for 10 min in TBS containing 1% tween (TBST). Membranes were then blocked in 5% dry non-fat milk powder in TBST for 1 h and incubated overnight at 4°C with primary antibodies against AQP4 (Rabbit Anti-AQP4, AB3594; 1:800, Abcam, Cambridge, UK), Aβ (Rabbit anti-Aβ, AB10148; 1:200, Abcam, Cambridge, UK), β-tubulin (Chicken Anti- β-tubulin, #2,146; 1:1,000, Cell Signaling, Boston, MA, USA), and GAPDH (Rabbit Anti-GAPDH; AB2302, 1:1,000, Millipore Sigma, Burlington, MA, USA). Following three washes in TBST at respective concentrations, membranes were incubated for 1 h with HRP-conjugated secondary antibodies as follows: Goat Anti-Rabbit IgG (1:10,000 for AQP4, 1:500 for Aβ and 1:10,000 for GAPDH), and mouse anti-chicken IgG (1:20,000). Finally, membranes were washed three times with TBST. Immobilon Western Chemiluminescent HRP-substrate (Millipore Sigma, Burlington, MA, USA) and hydrogen peroxide were used in equal proportions to develop the image at a suitable exposure level until bands were clearly visible. Densitometry was performed using ImageJ software on each of the three proteins and normalized to GAPDH or β-tubulin, and the optical density measured and plotted for the hippocampus or cortex. AQP4 appeared in its isomeric form at ∼38 kDa and was compared with β-tubulin ∼50 kDa while GFAP ∼55 kDa was analyzed with GAPDH ∼35 kDa.

### Statistical analysis

All data were expressed as means ± SEM and tested for normality (Wilk–Shapiro’s test) and homoscedasticity (Leven’s test). Data were then analyzed using SPSS Software (SPSS Inc., USA). All analyses were performed using univariate and repeated-measures ANOVA and independent sample Student’s *t*-tests, as indicated. The significance level selected was α = 0.05 for all tests and was Bonferroni-corrected where appropriate.

## Results

### Chronic neuroinflammation impairs the clearance of fluorophore-conjugated dextran tracers

**FIGURE 2:**
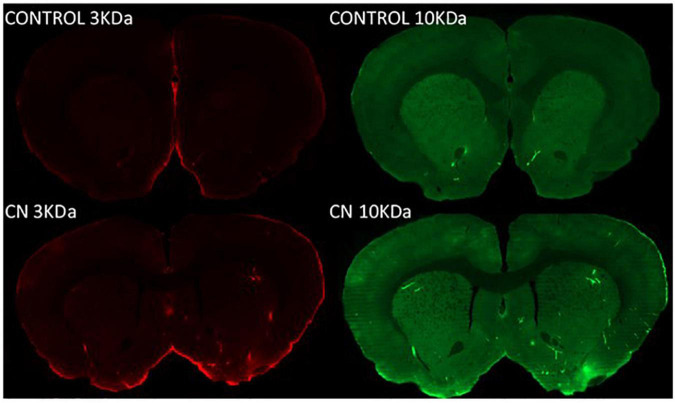
Parenchymal tracer distributions following 8 weeks of chronic Neuroinflammation (Cohort 1). Parenchymal distributions of Texas red-conjugated 3-kDa dextran (left) and FITC-conjugated 10-kDa dextran (right) tracers are shown at 30-min post-infusion in representative coronal section images from Control (top row) and CN group (bottom row) brains. Parenchymal distributions of both tracers were increased in the CN group relative to Control.

**FIGURE 3:**
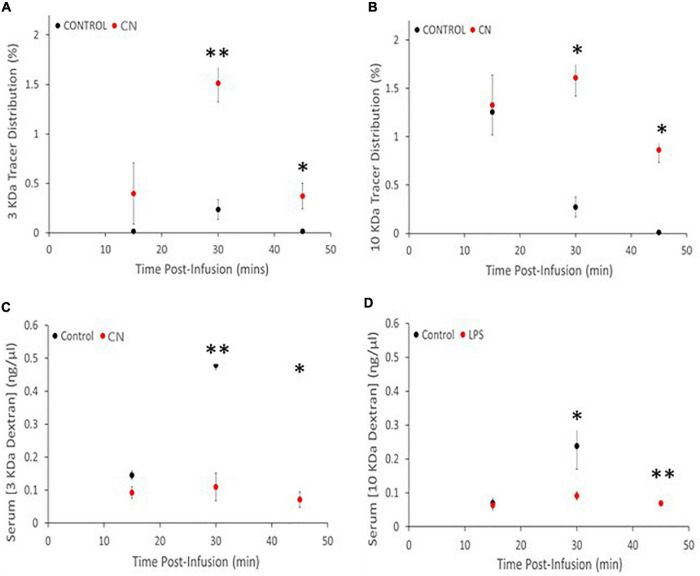
Chronic neuroinflammation impairs tracer clearance from brain parenchyma in the CN group relative to Control. **(A,B)** Parenchymal distributions of 3-kDa and 10-kDa dextran tracers were comparable for the CN and Control groups, reflecting efficient tracer entry and/or accumulation. **(C,D)** Parenchymal distributions of both 3- and 10-kDa dextran tracers were significantly increased at 30- and 45-min post-infusion. Serum concentrations of both 3- and 10-kDa dextran tracers were decreased at all three time-points relative to Controls, with significant decreases at 30- and 45-min post-infusion. *P < 0.05 and ^**^P < 0.01.

Both 3-kDa (Texas-Red) and 10-kDa (FITC) dextran tracers readily entered the brain and were present in both perivascular spaces and in the parenchyma in CN and Control group animals (Figure 2). Densitometric analysis of tracer distribution (10–12 slices per brain) did not reveal significant differences between the CN and Control groups following 15 min of tracer circulation (3-kDa, *t* = 1.47, *P* n.s.; 10-kDa, *t* = 0.07, *P* n.s.). However, parenchymal distributions of both tracers were significantly increased in the CN group relative to Controls following 30 min of circulation (3-kDa, *t* = 5.92, *P* < 0.01; 10-kDa, *t* = 4.54, *P* < 0.05), and remained significantly increased following 45 min of circulation (3-kDa, *t* = 2.78, *P* < 0.05; 10-kDa, *t* = 3.29, *P* < 0.05) (Figure 3).

Spectrophotometric analysis of tracer concentrations in the serum revealed an inverse relationship to these parenchymal tracer distributions. There were no significant differences in serum tracer concentrations between CN and Control groups following 15 min of tracer circulation (3-kDa, *t* = 2.46, *P* < 0.10; 10-kDa, *t* = 0.35, *P* n.s.). However, serum concentrations of both tracers were significantly reduced in the CN group relative to Control following 30 min of circulation (3-kDa, *t* = 5.36, *P* < 0.01; 10-kDa, *t* = 2.94, *P* < 0.05), and remained significantly reduced following 45 min of circulation (3-kDa, *t* = 5.49, *P* < 0.05; 10-kDa, *t* = 6.33, *P* < 0.01) (Figure 3).

### Chronic neuroinflammation increases amyloid β within the hippocampus and promotes perivascular deposits of amyloid β within penetrating vessels

**FIGURE 4:**
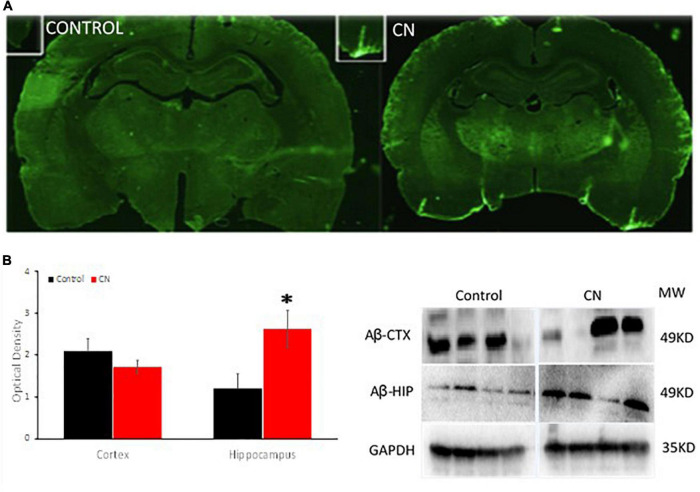
Chronic neuroinflammation significantly increases levels of Aβ within the hippocampus and promotes perivascular amyloid β deposits. **(A)** Aβ distribution in coronal sections from Control (left) and CN (right) group brains. Relative to Controls, chronic neuroinflammation causes an apparent increase in levels of Aβ within the perivascular space of penetrating vessels from the pial membrane and within the adjacent brain parenchyma. **(B)** Semi-quantitative analysis of Aβ expression within the pre-frontal cortex and hippocampus with a representative Western blot, normalized using GAPDH. Relative to Controls, chronic neuroinflammation significantly increased levels of Aβ in the hippocampus, whereas Aβ levels were not significantly affected in the pre-frontal cortex. This pattern of Aβ distribution is consistent with impaired clearance of this protein under these conditions. *P < 0.05.

Western blots using anti-Aβ antibodies were used to probe ∼49-kDa protein bands in lysates from the hippocampus and pre-frontal cortex. Amyloid β levels in the CN group were significantly increased within the hippocampus relative to Controls, whereas an analogous increase in Aβ expression was not observed in the pre-frontal cortex (cortex, *t* = 1.11, *P* n.s; hippocampus, *t* = 2.53, *P* < 0.05) (Figure 4). Immunofluorescence labeling for Aβ 1–42 did not reveal evidence of Aβ plaques within the pre-frontal cortex or hippocampus in either CN- or Control-group brains. However, enhanced Aβ labeling was present in the CN group relative to Controls, localized to penetrating vessels extending from the pial surface. Higher magnification images of these vessels revealed punctate Aβ deposits within the perivascular space in the CN brains which were absent in Controls (Figure 4).

### Chronic neuroinflammation disrupts aquaporin-4 polarization on astrocytic endfeet processes

**FIGURE 5:**
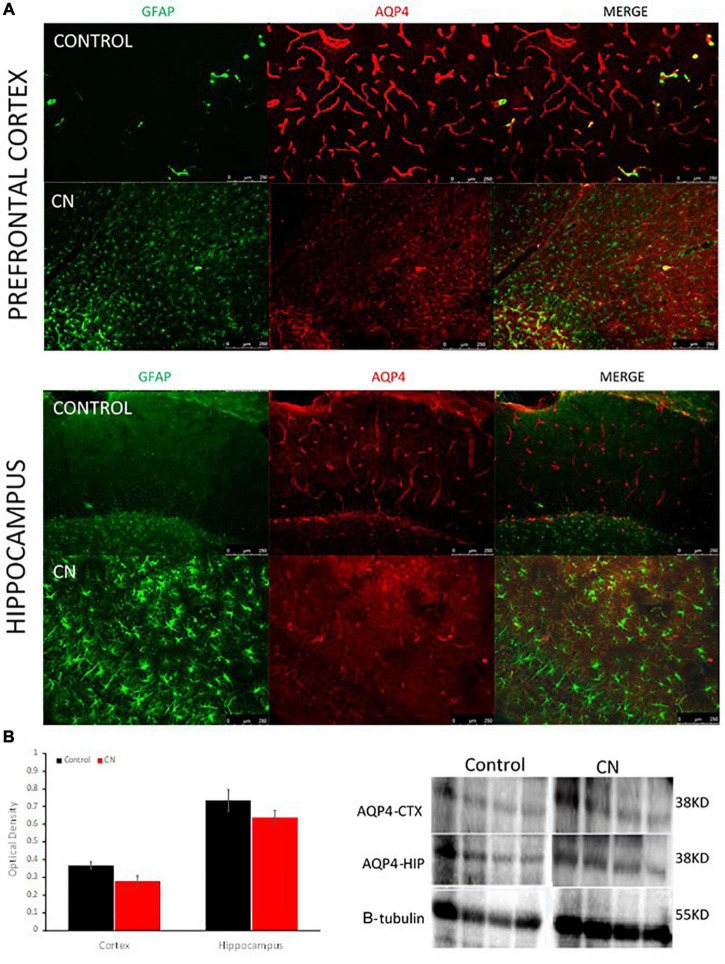
Chronic neuroinflammation disrupts aquaporin polarization on astrocytic endfeet processes. **(A)** Representative double-label immunofluorescence images of pre-frontal cortex and hippocampus from Control- and CN-group brains. In Controls, astrogliosis was nominal and AQP4s were highly polarized on astrocytic endfeet surrounding cerebral blood vessels. In the CN-group, astrogliosis was pronounced and AQP4s were largely depolarized within these structures (right, merged) and elsewhere within the brain. **(B)** Semi-quantitative analysis of AQP4 expression within the pre-frontal cortex and hippocampus with a representative Western blot, normalized using beta tubulin. AQP4 expression was not significantly affected within these regions in the CN group, relative to Control. Thus, the reduction of aquaporin polarization in the CN group appears to primarily reflect a redistribution of aquaporins across the astrocyte surface and away from perivascular endfeet processes.

Western blots using anti-AQP4 antibodies were used to probe ∼38-kD protein bands in lysates from the hippocampus and pre-frontal cortex. Our antibody detected a protein band of ∼ 38-kDa, the M1 form of the protein comprised of 323 amino acids. A downward trend in AQP4 expression was apparent in both the hippocampus and pre-frontal cortex in the CN group relative to Controls; however, this did not reach statistical significance in either region (cortex, *t* = 2.23, *P* n.s.; hippocampus, *t* = 0.66, *P* n.s.) (Figure 5). Immunofluorescence labeling for AQP4 within the hippocampus and pre-frontal cortex revealed multiple regions where aquaporin polarization on astrocytic endfeet processes was disrupted in the CN group relative to Controls. Double-label immunofluorescence revealed that regions with disrupted AQP4 polarization frequently coincided with regions where reactive astrocytes were densely populated (Figure 5). This was more apparent at higher magnifications where GFAP-positive astroglial processes were routinely observed proximal to and encircling blood vessels in the CN group (Supplementary Figure 1). GFAP-positive reactive astrocytes were prevalent within multiple brain regions the CN group, consistent with a chronic neuroinflammatory state, whereas these were virtually absent in Controls (Supplementary Figure 2). Importantly, astrocyte activation cannot be induced by LPS and is instead induced and maintained by activated microglial cytokine production (Liddelow et al., 2017).

### Chronic neuroinflammation selectively enhances contextual fear memory

**FIGURE 6:**
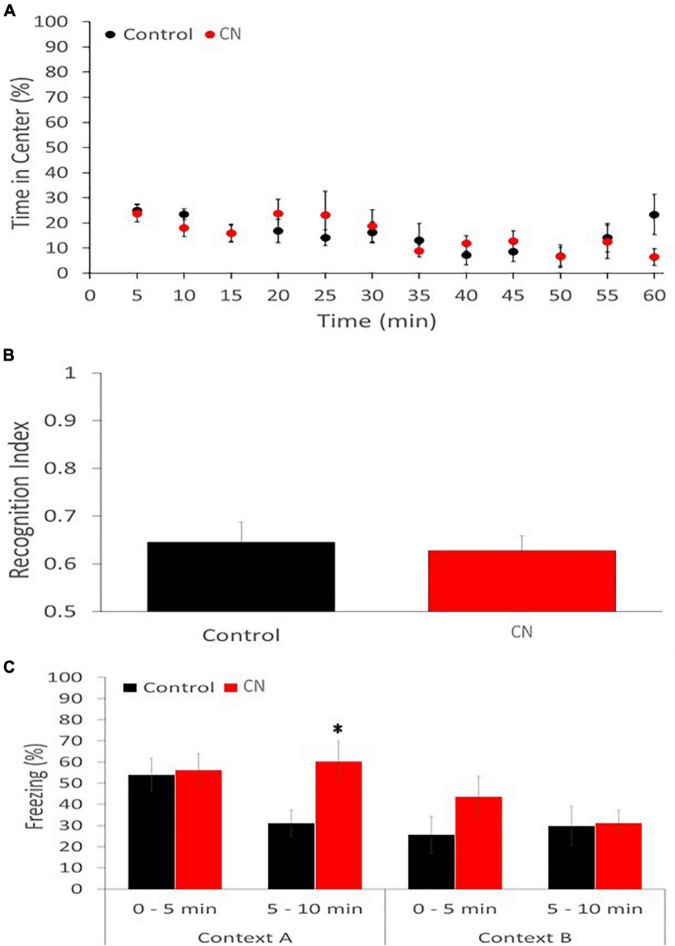
Chronic neuroinflammation impairs extinction of contextual fear memory. **(A)** Open-field behavior was not significantly affected in the CN group relative to Controls during a 60-min monitoring session, including the percentage of time spent in the center of the field. **(B)** Novel object recognition memory was similarly not impaired in the CN group relative to Control, as reflected by the recognition index. **(C)** Contextual fear conditioning memory was significantly enhanced in the CN group relative to Control. Specifically, freezing time in Context A, during the 5–10-minuite epoch, was significantly increased relative to Controls but not in the 0–5-min epoch. A trend toward increased freezing during the 0–5-min epoch in Context B was also apparent, consistent with context generalization. *P < 0.05.

The open-field assay revealed no significant difference between the CN and Control group with respect to the time spent in the center of the field vs. the periphery (Repeated-measures ANOVA: *F*_1,11_ = 1.194, *P* n.s.), suggesting the absence of anxiolytic effects related to chronic neuroinflammation (Figure 6A). Similarly, there was no significant difference between CN- and Control-groups with respect to the distance traveled in either the periphery or the center zones (data not shown).

The novel object recognition assay also failed to reveal a significant difference between the CN- and Control-groups with respect to the recognition index (Univariate ANOVA: *F*_1_ = 0.104, *P* n.s.), suggesting that recognition memory was unaffected by chronic neuroinflammation (Figure 6B).

The contextual fear-conditioning assay revealed a subtle group difference. On day 1, the CN- and Control-groups performed comparably during the acquisition of the shock-context association. When returned to Context A, 24-h after the shock-context association was formed, both groups of animals exhibited freezing behavior. However, while there was no significant difference in freezing times between CN and Control groups during the first 5-min epoch (*t* = 0.21, *P* n.s.), the CN group exhibited significantly greater freezing times relative to Control during the second 5-min epoch (*t* = 2.53, *P* < 0.05). This suggests that chronic neuroinflammation may have impaired the extinction of fear-induced contextual memory. When exposed to context B, 48-h after the shock-context association was acquired, the CN group also tended to exhibit greater freezing times relative to Controls during the first 5-min epoch (*t* = 1.38, *P* < 0.2), suggesting a trend toward enhanced context generalization related to chronic neuroinflammation. During the second 5-min epoch in Context B, freezing behavior was comparable between the two groups (*t* = 0.13, *P* n.s.) (Figure 6C).

## Discussion

Our results support our original hypothesis that chronic neuroinflammation is sufficient to impair the clearance of macromolecular waste from the brain parenchyma. Relative to age-matched controls, the clearance kinetics of both dextran tracers were significantly impaired by chronic neuroinflammation. At 15-min post-infusion into the cisterna magna, both tracers were visible within perivascular space and their respective distributions within the parenchyma were comparable for the CN and Control groups. However, at both 30- and 45-min post-infusion, parenchymal tracer distributions were significantly higher in the CN group relative to Controls, whereas tracer concentrations in the serum were significantly lower in the CN group at these time-points. Impaired waste clearance in the CN group was associated with significantly elevated levels of Aβ within the hippocampus relative to Controls, accompanied by perivascular deposits of Aβ within penetrating vessels near the pial surface. As expected, GFAP-positive reactive astrocytes were virtually absent in Controls but were prevalent within many brain regions in the CN group, including the hippocampus and pre-frontal cortex, accompanied by a corresponding disruption of AQP4 polarization on astrocytic endfeet processes. These pathologies were associated with significant increases in the persistence of contextual fear memory relative to age-matched Controls.

Dextran tracers within the CSF enter the parenchyma by traversing ∼20 ηm clefts between overlapping astrocytic endfeet, whereas the aqueous component of CSF is also able to enter through intracellular space *via* AQP4 channels (Kim et al., 2020; Alshuhri et al., 2021). Previous reports suggest that 3 kDa dextran tracers pass freely through these clefts whereas the passage of 40 kDa dextran tracers is impeded (Xavier et al., 2018). We reasoned that a similar disparity might exist between our 3- and 10-kDa dextran tracers in the chronically inflamed brain, perhaps reflecting a reduction in cleft size and/or the disruption of AQP4 polarization on astrocytic endfeet processes. However, although we saw clear evidence of activated astrocytes and AQP4 depolarization throughout the chronically inflamed brain, these did not appear to impede the entry of either tracer into the parenchyma. Recent imaging studies have revealed that the movement of CSF through intracellular space is the primary driver of convective bulk flow within the parenchyma and is highly dependent on AQP4 conduction (Asgari et al., 2015; Alshuhri et al., 2021). Thus, AQP4 depolarization in the chronically inflamed brain may have been sufficient to reduce the bulk flow of CSF/ISF and slow the glymphatic clearance of our dextran tracers once they had entered the parenchyma. Cellular hypertrophy associated with chronic neuroinflammation may have also reduced the volume of interstitial space, thereby impeding the movement of dextrans through the parenchyma *via* convective bulk flow.

Although chronic neuroinflammation did not impair the entry of either of our dextran tracers into the parenchyma, there were significant size-dependent differences in their kinetic profiles in both the CN and Control groups. This is inconsistent with bulk flow and suggests that these tracers move primarily by diffusion within the parenchyma, as proposed by the IPAD hypothesis (Smith et al., 2017; Albargothy et al., 2018). The IPAD hypothesis suggests that as tracers enter the parenchyma from the CSF, they diffuse toward the capillary before entering the IPAD clearance pathway (Morris et al., 2016). Since the rate of diffusion is inversely proportional to tracer size, smaller and more mobile tracers should move efficiently toward the capillary for clearance whereas larger tracers may distribute more broadly within the parenchyma prior to being cleared (Smith et al., 2017). This interpretation is consistent with the kinetics we observed in Controls, where 3-kDa tracers are cleared efficiently to the serum with minimal parenchymal distribution whereas the clearance of 10-kDa tracers to the serum is more gradual with significantly higher parenchymal distribution at 15-min post-infusion. When IPAD clearance is compromised, however, the rates of diffusion toward the capillary should be reduced for all tracers, thereby increasing their parenchymal distributions and decreasing their rates of deposition in the serum (Smith et al., 2017). This interpretation is consistent with the kinetics we observed in the CN group, where the parenchymal distributions of both the 3- and 10-kDa dextran tracers were significantly increased, and their rates of clearance and deposition in the serum were significantly impaired, relative to their respective Controls. Chronic neuroinflammation may have impaired IPAD clearance of both tracers by compromising vascular smooth muscle function and/or by impeding their movement within the capillary or tunica media basement membranes (Morris et al., 2016; Albargothy et al., 2018; Garcia-Miguel et al., 2018; Aldea et al., 2019). Cellular hypertrophy associated with chronic neuroinflammation may have also slowed the rates of diffusion of these tracers within the parenchyma.

The elevated levels of Aβ we observed in the chronically inflamed brain, combined with perivascular deposits of Aβ protein in penetrating vessels, are consistent with the early stages of cerebral amyloid angiopathy (Keable et al., 2016). These pathologies are commonly associated with Alzheimer’s disease and lend additional support for the IPAD interpretation of our data (Kim et al., 2020). The accumulation of Aβ along with other forms of macromolecular waste within the capillary and/or tunica media basement membranes has been suggested to trigger a cascade effect, leading to the toxic deposition of insoluble Aβ plaques and the gradual impairment of neuronal and cognitive function (Weller et al., 2015).

The effects of chronic neuroinflammation on behavioral measures of cognitive function were remarkably subtle relative to the effects associated with waste clearance. Using the same model of chronic neuroinflammation, Bossù et al. (2012) previously reported significant cognitive deficits in the open- field-, object- place-, and novel-object-recognition assays conducted at similar latencies. However, they administered these assays in combination whereas our open-field and novel-object-recognition assays were performed separately. Thus, it is possible that the environmental and/or task complexity associated with their approach was more suitable to detect these deficits. They did not detect deficits using the elevated-plus and Morris-water- maze assays, suggesting that anxiety and spatial memory, respectively, were unaffected under these conditions (Bossù et al., 2012). The relative persistence of contextual fear memory associated with chronic neuroinflammation is consistent with an enhanced state of arousal relative to age-matched Controls (Poulous et al., 2016, Chaaya et al., 2018).

Overall, our data reveal a novel association between chronic neuroinflammation and impaired waste clearance in the brain; however, there are important questions that remain unanswered. Given disparate reports regarding the role of aquaporins in this context, further investigations are required to determine whether and under what conditions the loss of aquaporin density impacts CSF and/or solute transport across the BBB. We did not perform immunofluorescence assays for AQP4 coincident with our tracer assays to avoid confounds related to the presence of FITC- and Texas-red-conjugated tracers within perivascular space, although previous reports suggest that reduced aquaporin density should have been well established at this time-point (Qin et al., 2007; Bossù et al., 2012). Similarly, we did not investigate whether vascular smooth muscle cells are impacted in our experimental model of chronic neuroinflammation. However, previous reports suggest that TNFα is sufficient to induce dedifferentiation and phenotypic switching of vascular smooth muscle cells, resulting in a downregulation of contractile proteins (Garcia-Miguel et al., 2018). Finally, we did not investigate whether chronic neuroinflammation may have impacted the clearance of tracers directly from the subarachnoid CSF, *via* the arachnoid villi and/or the meningeal lymphatic pathway. Since our tracers were infused directly into the subarachnoid CSF, clearance *via* these pathways was likely reflected to some extent in the clearance kinetics related to the serum (Welch and Pollay, 1961; Aspelund et al., 2015; Louveau et al., 2015, 2018; Ahn et al., 2019; Kutomi and Takeda, 2020). However, the inverse correlation between the parenchymal and serum clearance kinetics for both tracers suggests strongly our data primarily reflects clearance from the parenchyma. These unresolved issues are the focus of ongoing investigations in our laboratory.

## Conclusion

This study establishes that chronic neuroinflammation is sufficient to impair the clearance of macromolecular waste from the brain parenchyma and its removal to the serum, resulting in a pathological accumulation of Aβ within the hippocampus over time. Since tracer entry into the brain parenchyma was seemingly unaffected by AQP4 depolarization, and since there were size-dependent differences in tracer clearance kinetics in both the CM and Control groups, our data seem most consistent with the IPAD hypothesis. These results implicate chronic neuroinflammation as an initiator in the pathogenesis of vascular dementia and Alzheimer’s disease, and suggest that therapies targeting chronic neuroinflammation may mitigate the pathogenesis associated with these diseases following various forms of neurotrauma and/or in the aging brain.

## Data availability statement

The raw data supporting the conclusions of this article will be made available by the authors, without undue reservation.

## Ethics statement

The animal study was reviewed and approved by the Central Michigan University: Institutional Animal Care and Use Committee.

## Author contributions

KJ directed the research and designed the experiments with assistance from SS, developed the tracer injection and serum analysis protocols, and performed the blood draws and perfusions. JL developed the spectrophotometry protocol and performed the spectrophotometric serum analysis. SS performed the LPS injections, tracer-injection surgeries, immunohistochemistry, Western blot, microscopy, behavioral assays, and statistical analysis. SS and KJ co-wrote the manuscript. All authors read and approved the manuscript.

## Supplementary material

Supplementary Figure 1 Chronic neuroinflammation was confirmed by the presence of reactive astrocytes throughout the brain. Representative images depict the persistence of hypertrophic GFAP+ reactive astrocytes within the pre-frontal cortex at 36-weeks post-induction in the CN group, whereas reactive astrocytes were virtually absent in Controls. Astrocyte activation is induced and maintained by activated microglial cytokine production and cannot be induced by LPS (Liddelow et al., 2017).

Supplementary Figure 2 Higher magnification images of AQP4 and GFAP double-labeling. AQP4s (red) were highly concentrated on astrocytic endfeet processes in Controls with nominal astrocytic GFAP expression (green). AQP4s (red) were much less concentrated on astrocytic endfeet processes in the chronically inflamed brain and reactive astrocytes with enhanced GFAP expression (green) were prevalent. Intriguingly, reactive astrocytes commonly extended processes which encircled microvessels within these regions.
