# Supplementary figures and images for: Chronic neuroinflammation impairs waste clearance in the rat brain
Source: Front Neuroanat. 2022 Dec 7;16:1013808. doi: 10.3389/fnana.2022.1013808 (PMC9768431; doi:10.3389/fnana.2022.1013808)

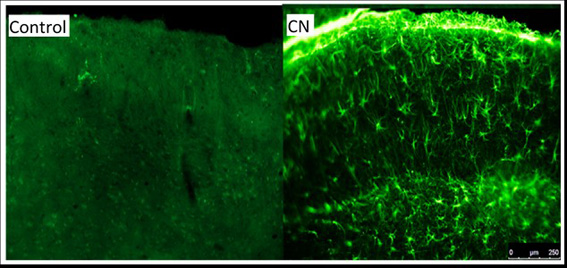

Supplement: Supplementary Figure 1 — Chronic neuroinflammation was confirmed by the presence of reactive astrocytes throughout the brain. Representative images depict the persistence of hypertrophic GFAP+ reactive astrocytes within the pre-frontal cortex at 36-weeks post-induction in the CN group, whereas reactive astrocytes were virtually absent in Controls. Astrocyte activation is induced and maintained by activated microglial cytokine production and cannot be induced by LPS (Liddelow et al., 2017). [file Image_1.JPEG]

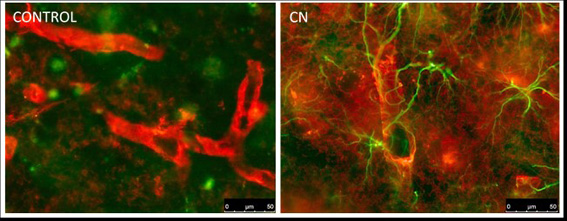

Supplement: Supplementary Figure 2 — Higher magnification images of AQP4 and GFAP double-labeling. AQP4s (red) were highly concentrated on astrocytic endfeet processes in Controls with nominal astrocytic GFAP expression (green). AQP4s (red) were much less concentrated on astrocytic endfeet processes in the chronically inflamed brain and reactive astrocytes with enhanced GFAP expression (green) were prevalent. Intriguingly, reactive astrocytes commonly extended processes which encircled microvessels within these regions. [file Image_2.JPEG]
